# miR-636: A Newly-Identified Actor for the Regulation of Pulmonary Inflammation in Cystic Fibrosis

**DOI:** 10.3389/fimmu.2019.02643

**Published:** 2019-11-15

**Authors:** Pauline Bardin, Tobias Foussignière, Nathalie Rousselet, Carine Rebeyrol, Joanna C. Porter, Harriet Corvol, Olivier Tabary

**Affiliations:** ^1^Faculté des Sciences, Sorbonne Université, Paris, France; ^2^Inserm, Centre de Recherche Saint-Antoine, Paris, France; ^3^UCL Respiratory, University College London, Hospitals NHS Foundation Trust, London, United Kingdom; ^4^Département de Pédiatrie Respiratoire, Hôpital Trousseau, AP-HP, Paris, France

**Keywords:** inflammation, airway, cystic fibrosis, miRNA, IL1R1, RANK, IKKβ

## Abstract

Cystic fibrosis (CF) results from deficient CF transmembrane conductance regulator (CFTR) protein activity leading to defective epithelial ion transport. Pulmonary degradation due to excessive inflammation is the main cause of morbidity and mortality in CF patients. By analysing miRNAs (small RNAseq) in human primary air-liquid interface cell cultures, we measured the overexpression of miR-636 in CF patients compared to non-CF controls. We validated these results in explant biopsies and determined that the mechanism underlying miR-636 overexpression is linked to inflammation. To identify specific targets, we used bioinformatics analysis to predict whether miR-636 targets the 3′-UTR mRNA regions of *IL1R1* and *RANK* (two pro-inflammatory cytokine receptors), *IKBKB* (a major protein in the NF-κB pathway), and *FAM13A* (a modifier gene of CF lung phenotype implicated in epithelial remodelling). Using bronchial epithelial cells from CF patients to conduct a functional analysis, we showed a direct interaction between miR-636 and *IL1R1, RANK*, and *IKBKB*, but not with *FAM13A*. These interactions led to a decrease in IL1R1 and IKKβ protein expression levels, while we observed an increase in RANK protein expression levels following the overexpression of miR-636. Moreover, NF-κB activity and IL-8 and IL-6 secretions decreased following the transfection of miR-636 mimics in CF cells. Similar but opposite effects were found after transfection with an antagomiR-636 in the same cells. Furthermore, we demonstrated that miR-636 was not regulated by *Pseudomonas aeruginosa* in our model. We went on to show that miR-636 is raised in the blood neutrophils, but not in the plasma, of CF patients and may have potential as a novel biomarker. Collectively, our findings reveal a novel actor for the regulation of inflammation in CF, miR-636, which is able to reduce constitutive NF-κB pathway activation when it is overexpressed.

## Introduction

Pulmonary degradation due to excessive inflammation is the main cause of morbidity and mortality in cystic fibrosis (CF) patients. CF is caused by a CF transmembrane conductance regulator (CFTR) gene mutation that encodes a chloride channel (1). Compromised activity of this channel at the apical membrane of bronchial epithelial cells leads to ionic imbalance and thickened mucus (2). The resulting mucosal hyper-viscosity promotes bacterial colonisation, notably by *Pseudomonas aeruginosa*, and establishes infection/inflammation cycles, resulting in the degradation of the pulmonary epithelium (3). Bronchial inflammation occurs at an early stage and contributes to the prolonged development of severe CF disease. This inflammation is a key point in the evolution of CF pathology, which is characterised by massive polymorphonuclear neutrophil (PMN) invasion at the inflammatory site due to IL-8 chemo-attraction secreted by pulmonary epithelial cells (4). PMNs, the overproduction of elastase, and IL-8 hypersecretion in the airway are the inflammatory hallmarks of CF lung disease (5, 6). IL-8 secretion is constitutively induced by bronchial epithelial cells and also stimulated by bacterial lipopolysaccharides, tumour necrosis factor-α (TNF-α), or interleukin-1β (IL-1β) through the activation of signalling pathways, such as NF-κB by the receptors interleukin 1 receptor 1 (IL1R1) (7–10) or TNF receptor sub-family 11A (TNFRSF11A, also called RANK). The receptor activator of nuclear factor κB ligand (RANKL) and its receptor RANK are part of the TNF and TNF receptor families, respectively, TNFSF11, RANK, and TNFRSF11B (11). Additionally, IL-1β is able to activate another target—*FAM13A* (FAMily with sequence similarity 13 member A), a modifier gene of CF. FAM13A promotes epithelial-mesenchymal transition and consequently remodelling in CF epithelial cells compared to the cells of healthy subjects (12). Despite these discoveries, the origin of hyperinflammation in CF is not well-understood, although microRNAs (miRNAs) are suspected to be involved.

miRNAs are small endogenous non-coding single-stranded RNA molecules that negatively regulate gene expression. A miRNA can act on the 3′-UTR (untranslated region) of mRNA, leading to its inhibition or degradation (13). Moreover, miRNAs regulate more than 60% of human protein-coding genes, affecting many physiological functions (14). For this reason, miRNAs play a critical role in many diseases characterised by the dysregulation of their expression. Certain studies have focused on the role of miRNA in regulating *CFTR* gene expression (15, 16) and others on the regulation of inflammatory processes (17). The role of miR-199a-3p in the negative regulation of NF-κB pathway activation through IKKβ has been previously examined (18).

In this study, we aimed to understand the role of miR-636, a miRNA we found dysregulated in the context of CF (18), on the regulation of inflammation in CF patients. We assessed miRNA and mRNA expression in air-liquid interface (ALI) cell cultures and in bronchial samples from CF patients and non-CF healthy subjects. We also performed experimental *in vitro* modulation of miR-636 expression to elucidate the regulation of four different targets (IL1R1, RANK, IKKβ, and FAM13A), determined by bioinformatics analysis and confirmed by functional analysis, in the context of CF. Finally, we determined a potential role for miR-636 neutrophil and plasma biomarkers of inflammation in CF patients.

## Materials and Methods

### Human Bronchial Epithelial Cell Culture

The human bronchial epithelial cell line CFBE41o- (CF) was a gift from Prof. DC Gruenert (UCSF, San Francisco, CA, USA). Cells were cultured in minimum essential medium (MEM) in the presence of Earle's salts and L-glutamine (Thermo Fisher Scientific, Villebon-Sur-Yvette, France) containing 10% bovine growth serum (Eurobio, Les Ulis, France) and 1% penicillin/streptomycin (Thermo Fisher Scientific). Cell cultures were grown and maintained at 37°C in a 5% CO_2_ humidified incubator. All cells were tested for mycoplasma contamination (Lonza, Ambroise, France).

Human bronchial epithelial cells isolated from bronchial biopsies from five CF (F508del/F508del) patients and non-CF healthy donors were purchased from Epithelix SARL (Geneva, Switzerland) (19). The cells were fully differentiated in air-liquid interface (ALI) cultures (MucilAir™) according to the provider's recommendations.

### Human Lung Explants, Plasma, and Neutrophils

Human lung explants provided by Dr. S. Blouquit-Laye (UVSQ, Versailles, France) were collected and processed in compliance with the standard guidelines for human research (Declaration of Helsinki) and with current French public health legislation (L.1235-2 and L.1245.2 articles, http://www.legifrance.gouv.fr). Each participating institution informed patients and ensured that they were not opposed to the use of surgical samples, removed during a medical act, for research purposes, and written informed consent was obtained from the participants of this study. Lung fragments were obtained from 14 non-CF controls undergoing surgery (45 ± 21 years old) and from 16 CF patients (F508del/F508del; 35 ± 9 years old) undergoing lung transplantation. For non-CF controls, samples were obtained from a non-pathological area without inflammatory cells from patients with bronchial carcinoma. After tissue dissection, samples were frozen immediately in liquid nitrogen before miRNA/RNA extraction.

Plasma samples were collected after obtaining informed consent from each patient included in the study during annual blood tests from 18 non-CF controls (30 ± 13 years old) and 17 CF patients (F508del/F508del; 15 ± 3 years old). The blood samples were centrifuged for 15 min at 3,000 *g*. The plasma (supernatant) was harvested, aliquoted, and stored at −80°C until miRNA extraction.

Blood was collected from eight non-CF controls (31 ± 6 years old) and six CF patients (F508del/F508del; 12 ± 2 years old) at UCLH NHS Trust, London, UK. Neutrophils were isolated by Percoll density centrifugation from sodium citrate anticoagulated blood before aliquoting and storage at −80°C. Ethical approval was provided by the UK National Research Ethics Committee (13/LO/0900) and a material transfer agreement was secured.

### Predicted miRNA Target

The role of miR-636 in the regulation of protein expression was examined using a computational approach that predicted the 3′-UTRs of *IL1R1, RANK, IKBKB*, and *FAM13A* mRNA to contain seed regions that are recognised by a variety of miRNAs including miR-636 (Table 1). We used several online algorithms: miRWalk (http://zmf.umm.uni-heidelberg.de/apps/zmf/mirwalk2/), miRanda (http://www.microrna.org), RNA22 (https://cm.jefferson.edu/rna22/Interactive/), and Targetscan (http://targetscan.org). The NCBI (https://www-ncbi-nlm-nih-gov.gate2.inist.fr/pubmed?holding=ifrinsblib) and e!Ensembl genome browsers (http://www.ensembl.org/index.html) provided information on the human *IL1R1, RANK, IKBKB*, and *FAM13A* transcripts, and miRBase (http://www.mirbase.org) provided information on miR-636.

**Table 1:** Summary table of the mRNA predicted to be targeted by miR-636 from different databases.

**Genes**	**EntrezID**	**REfseqID**	**miRNA**	**miRWalk**	**miRanda**	**RNA22**	**Targetscan**	**Sum**
*IL1R1*	3554	XM_005263933	hsa-miR-636	1	1	1	1	4
*RANK*	8792	NM_003839		1	0	1	1	3
*IKBKB*	3551	NM_001190720		1	0	0	1	2
*FAM13A*	10144	NM_014883		1	1	1	1	4

### Cell Transfection

CFBE41o- cell lines were transfected with miR-636 mimic (mirVana® miRNA mimic), mimic control (mirVana® miRNA mimic control), antagomiR-636 (mirVana® miRNA inhibitor), or antagomiR control (mirVana® miRNA inhibitor control) (Thermo Fisher Scientific, Courtaboeuf, France) using HiPerFect® (30 nM; Qiagen, Les Ulis, France) according to the manufacturer's instructions. The mature miR-636 sequence is 5′-UGUGCUUGCUCGUCCCGCCCGCA-3′. Forty-eight hours after transfection, the supernatants were recovered, and the cells were lysed for miRNA, RNA, and protein extractions.

### *Pseudomonas aeruginosa* Stimulation

CFBE41o- cell lines were stimulated with PAK, a strain of *P. aeruginosa* heat-killed, at a 0.25 multiplicity of infection (MOI), or without (control condition) during 24 h. The strain was heated at 65°C in a water bath for 15 min to kill the bacteria without altering the virulence factors (e.g., LPS and flagellin), thus allowing the bacteria to infect our cells for longer periods of time, i.e., 24 h. Twenty-four hours after the stimulation, the supernatants were recovered and the cells were lysed for miRNA and RNA extractions.

### RNA and miRNA Extraction and RT-qPCR Analysis

For miRNA and RNA extraction from human lung explants, 100 mg of pulmonary tissue sample was crushed with a POLYTRON® probe (PT 3 100; Kinematica, Luzern, Switzerland) in 1 ml of TRIzol® (Life Technologies, Saint-Aubin, France) (20).

miRNA and RNA were extracted from cells using a Nucleospin miRNA kit (Macherey-Nagel, Düren, Germany). For plasma and neutrophil samples, miRNA was extracted using a Nucleospin miRNA Plasma kit (Macherey-Nagel). RNA and miRNA were eluted with 30 μL of sterile RNase-free water. The concentration and quality of the RNA and miRNA were evaluated using a DS-11 Series Spectrophotometer (DeNovix, Wilmington, DE, USA) based on absorbance at 260 nm.

miR-636, miR-103, miR-16-5p, and *RNU6B* were reverse-transcribed with a TaqMan™ MicroRNA Assay Kit (Thermo Fisher Scientific) using 20 ng of miRNA. *IL1R1, RANK, IKBKB, FAM13, IL-8, IL-6*, and *GAPDH* were reverse-transcribed with a High Capacity cDNA Reverse Transcription Kit (Thermo Fisher Scientific) using 1 μg of RNA. qPCR was performed using an ABI StepOnePlus™ (Thermo Fisher Scientific) and TaqMan™ technology. For relative quantification, miRNA and RNA levels were calculated using the 2^−ΔΔCT^ method and normalised to the expression levels of *RNU6B*, miR-103, miR-16-5p, and *GAPDH*. Each sample was assessed in triplicate.

### Luciferase Assay

For the luciferase assay, we used an *IL1R1*-3′-UTR wild-type (WT), *RANK*-3′-UTR WT, *IKBKB*-3′-UTR WT, and *FAM13A*-3′-UTR WT plasmid: CGCCCCCCTG and mutated plasmid GCGGGAGAC for all of the targets except for FAM13A (Tebu-bio, Le Perray en Yvelines, France). CF cells were seeded in six-well plates and transfected the next day with 1 μg/mL of each plasmid using an ExGen 500 (Euromedex, Souffelweyersheim, France). Cells were co-transfected with 30 nM miR-636 or a mimic control. Supernatants were collected 48 h after co-transfection. For these experiments, we used a Secrete-Pair™ Dual Luminescence Assay Kit (Genecopoeia, Tebu-bio, Rockville, MD, USA) designed to analyse the activities of Gaussia luciferase and secreted embryonic alkaline phosphatase (SEAP), as well as an endogenous reporter using the same samples from the cell culture medium.

### Protein Extraction and Western Blot Analysis

Proteins were extracted using a lysing solution (RIPA, anti-protease cocktail). Total protein concentration was evaluated with a Pierce™ BCA Protein Assay Kit (Thermo Fisher Scientific).

Next, 20 μg of total protein extract was reduced and size-separated on 10% SDS-polyacrylamide gels, transferred to nitrocellulose or PVDF membranes (Invitrogen, Paris, France), blocked in TBS-T milk, and incubated with specific primary antibodies against IL1R1 (# HPA005823 at 1:500), RANK (# PA80145 at 1:1,000), IKKβ (# 2884 at 1:1,000), and βactin (# 3700 at 1:10,000) (Cell Signaling Technology, Danvers, MA, USA). The proteins of interest were detected, imaged, and quantified (Chemidoc; Bio-Rad, Marnes-la-Coquette, France).

### NF-κB Activity

Nuclear proteins were extracted from cells transfected with miR-636 mimic, mimic control, antagomiR-636, or antagomiR control using a nuclear extract kit (Active Motif, Rixensart, Belgium). Total protein concentration was evaluated with a Qubit fluorometer (Thermo Fisher Scientific). The transcriptional activity of p65 NF-κB in 20 μg of nuclear protein extract from CFBE41o^-^ cells was assayed using a TransAM™ Transcription Factor Assay Kit (Active Motif).

### IL-8 and IL-6 Assay (ELISA)

The supernatants of cells transfected with miR-636 mimic, mimic control, antagomiR-636, or antagomiR control were recovered 48 h after transfection. IL-8 and IL-6 were quantified in each sample with the human IL-8 Duo-Set kit or human IL-6 Duo-Set kit (R&D Systems, Lille, France).

### Statistical Analysis

All data are described as mean ± SEM. Between-group differences were assessed using the Mann–Whitney *U*-test. In the figures, statistically significant differences are noted as *p* ≤ 0.05 (^*^), *p* ≤ 0.01 (^**^), and *p* ≤ 0.001 (^***^).

## Results

### Expression of miR-636, *IL1R1, RANK*, and *IKBKB* in CF and Non-CF ALI Cell Cultures

In our previous work, miR-636 expression was found to be significantly overexpressed in CF ALI cell cultures compared to non-CF cultures (18). Small RNAseq raw data files are available in the European Nucleotide Archive (ENA) (primary accession number ERP116236; http://www.ebi.ac.uk/ena/data/view/ERP116236).

**Figure 1:**
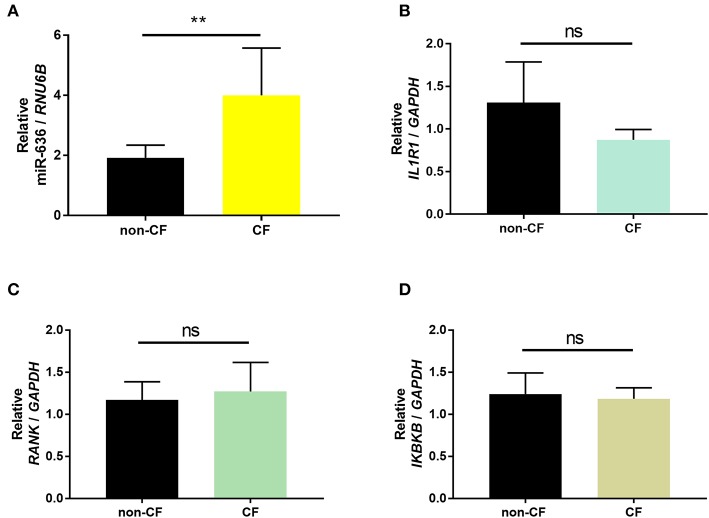
Validation of the results of small RNAseq and RNAseq. Quantification of miR-636 expression (**A**, *p* = 0.0043), *IL1R1* (**B**, *p* = 0.3929), *RANK* (**C**, *p* = 0.5714), and *IKBKB* (**D**, *p* = 0.5714) in bronchial epithelial cells from CF patients (*n* = 5) and healthy subjects (non-CF; *n* = 5) cultured at the air-liquid interface. A Mann–Whitney test was used to determine significance, ^**^p ≤ 0.01.

In order to validate the expression patterns obtained from small RNAseq and RNAseq, we conducted RT-qPCR on the samples to confirm expression of miR-636 and the mRNA targets (*IL1R1, RANK*, and *IKBKB)*. We observed a significant increase in miR-636 expression but no significant differences in *IL1R1, RANK*, and *IKBKB* mRNA expression in the CF ALI cell cultures compared to that in the non-CF cultures (Figure 1). These results validate those previously published for the small RNAseq and RNAseq (18).

### Expression Levels of miR-636, *IL1R1, RANK, IKBKB, FAM13A, Il-8*, and *IL-6* in CF and Non-CF Bronchial Tissues

To characterise and confirm the relevance of the selected targets in an *ex vivo* model, we quantified the expression levels of miR-636, *IL1R1, RANK, IKBKB*, and *FAM13A* and the two secreted cytokines, *IL-8* and *IL-6*, in human lung explants from CF patient and non-CF control biopsies using RT-qPCR.

**Figure 2:**
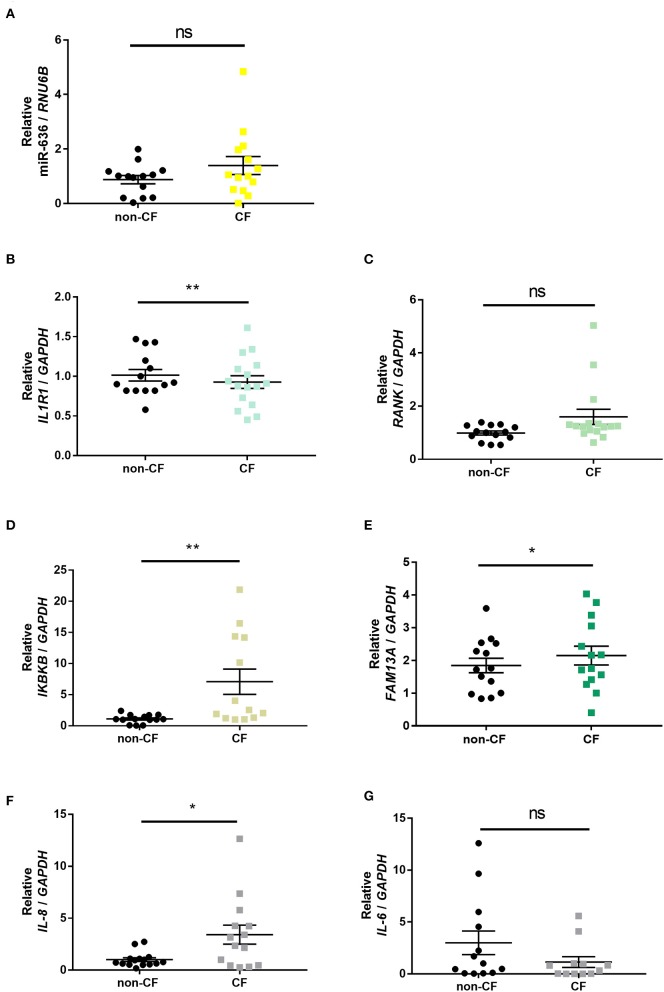
Expression of miR-636, *IL1R1, RANK, IKBKB, FAM13A, IL-8*, and *IL-6* in CF and non-CF bronchial explants. Quantification of the expression of miR-636 (**A**, *p* = 0.1781), *IL1R1* (**B**, *p* = 0.0024), *RANK* (**C**, *p* = 0.0925), *IKBKB* (**D**, *p* = 0.0053), *FAM13A* (**E**, *p* = 0.0141), *IL-8* (**F**, *p* = 0.0332), and *IL-6* (**G**, *p* = 0.1063) relative to *RNU6B* and *GAPDH*, respectively, in bronchial explants CF (*n* = 14) and non-CF (*n* = 16). A Mann–Whitney test was used to determine significance, ^*^p ≤ 0.05 and ^**^p ≤ 0.01.

We observed that all measured mRNA and miRNA targets were more heterogeneously expressed in CF bronchial tissues than in non-CF bronchial tissues (Figure 2). In CF patients, miR-636 and *RANK* appeared to be non-significantly overexpressed (Figures 2A,C); *IL1R1* mRNA levels were significantly lower (Figure 2B); *IKBKB, FAM13A*, and *IL-8* mRNA levels were significantly higher (Figures 2D–F), while *IL-6* mRNA seemed to be decreased (Figure 2G) compared to non-CF controls. Figures 2D,F confirmed and reinforced the data published previously (18).

### miR-636 Directly Regulates *IL1R1, RANK*, and *IKBKB* Expression Levels but Not *FAM13A*

In CF patients, we hypothesised that miR-636 is able to act on the four predicted targets, thereby influencing pulmonary inflammation. The CF cells were transfected with miR-636 at different concentrations (10, 30, 60, and 100 nM) or with mimic controls and cultured for 48 h (Figure S1A). miR-636 expression was analysed by RT-qPCR. The level of miR-636 in the presence of 30 nM of the mimic during 48 h increased by a factor of 10,000 compared to the control (Figure S1B). We also showed that the levels of endogenous miR-636 decreased by a factor of 40% in the presence of 30 nM of antagomiR-636 compared to that in the antagomiR control (Figure S1C). Having established these optimal conditions, subsequent experiments were performed with 30 nM of miR-636 mimic and/or antagomiR.

**Figure 3:**
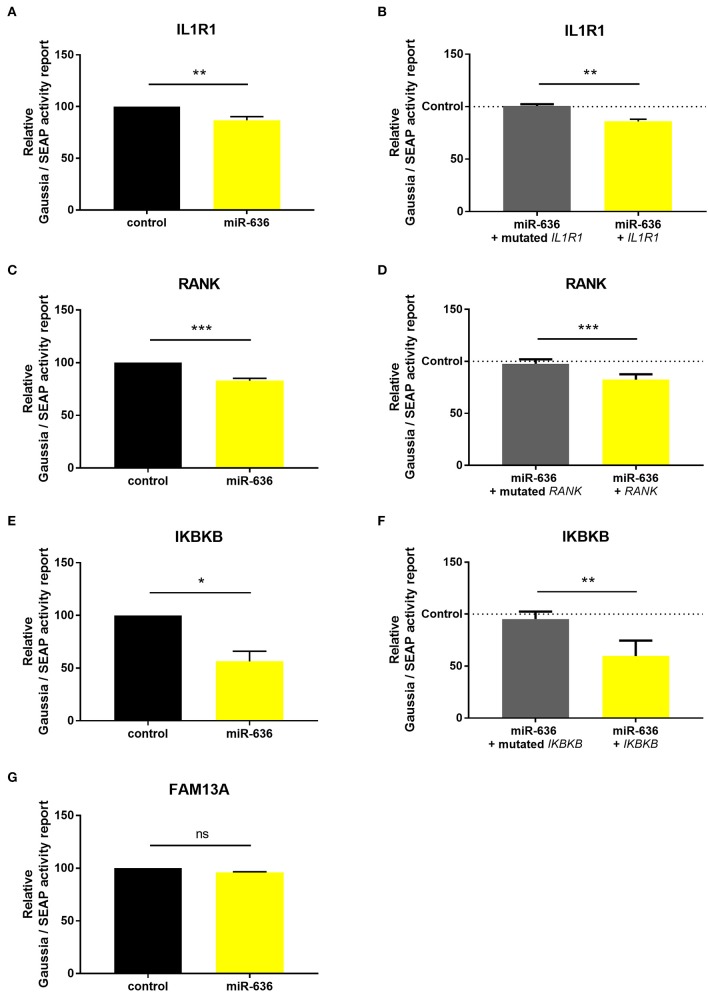
Study of the binding of miR-636 to 3′-UTR. Quantification of normalized luciferase activity by SEAP activity in CF cells (CFBE41o-) co-transfected with miR-636 mimic or a mimic control and a 3′-UTR luciferase plasmid of *IL1R1* (**A**, *p* = 0.0079), *RANK* (**C**, *p* < 0.0007), *IKBKB* (**E**, *p* = 0.0457), and *FAM13A* (**G**, *p* = 0.8950) 3′-UTR WT (*n* = 4) or 3′-UTR of *IL1R1* (**B**, *p* = 0.0013), *RANK* (**D**, *p* < 0.0007), and *IKBKB* (**F**, *p* = 0.0016) mutated (*n* = 5) over 48 h. A Mann–Whitney test was used to determine significance, ^*^p ≤ 0.05, ^**^p ≤ 0.01, and ^***^p ≤ 0.001.

To establish whether miR-636 modulates *IL1R1, RANK, IKBKB*, or *FAM13A* expression levels by binding to their 3′-UTRs, CF cells were co-transfected with a luciferase reporter vector containing the 3′-UTR region of WT mRNA (WT-IL1R1-3′UTR, WT-RANK-3′-UTR, WT-IKBKB-3′-UTR, and WT-FAM13A-3′-UTR) or with the 3′-UTR mutated region of each target except *FAM13A* (M-IL1R1-3′UTR, M-RANK-3′-UTR, and M-IKBKB-3′-UTR) and with the miR-636 mimic or mimic control. After 48 h, transfection with the mimic induced stronger miR-636 expression than did transfection with the control (Figure S1B). We observed a significant decrease in luciferase activity after co-transfection with the miR-636 mimic/WT-IL1R1-3′UTR compared to co-transfection with the mimic control/WT-IL1R1-3′UTR (Figure 3A); furthermore, this decrease was significant compared to that of the miR-636 mimic/M-IL1R1-3′UTR (Figure 3B). We observed the same results for *RANK* (Figures 3C,D) and *IKBKB* (Figures 3E,F). However, we observed no variation after 48 h of transfection with the miR-636 mimic/WT-FAM13A-3′-UTR compared to transfection with the mimic control/WT-FAM13A-3′-UTR (Figure 3G). For this reason, no experiment was performed with a M-FAM13A-3′UTR. These experiments confirm that miR-636 directly interacts with the 3′-UTR regions of *IL1R1, RANK*, and *IKBKB*, but not with that of *FAM13A*.

### miR-636 Represses IL1R1 and IKKβ Protein Expression and Increases RANK Protein Expression in CF Cells

**Figure 4:**
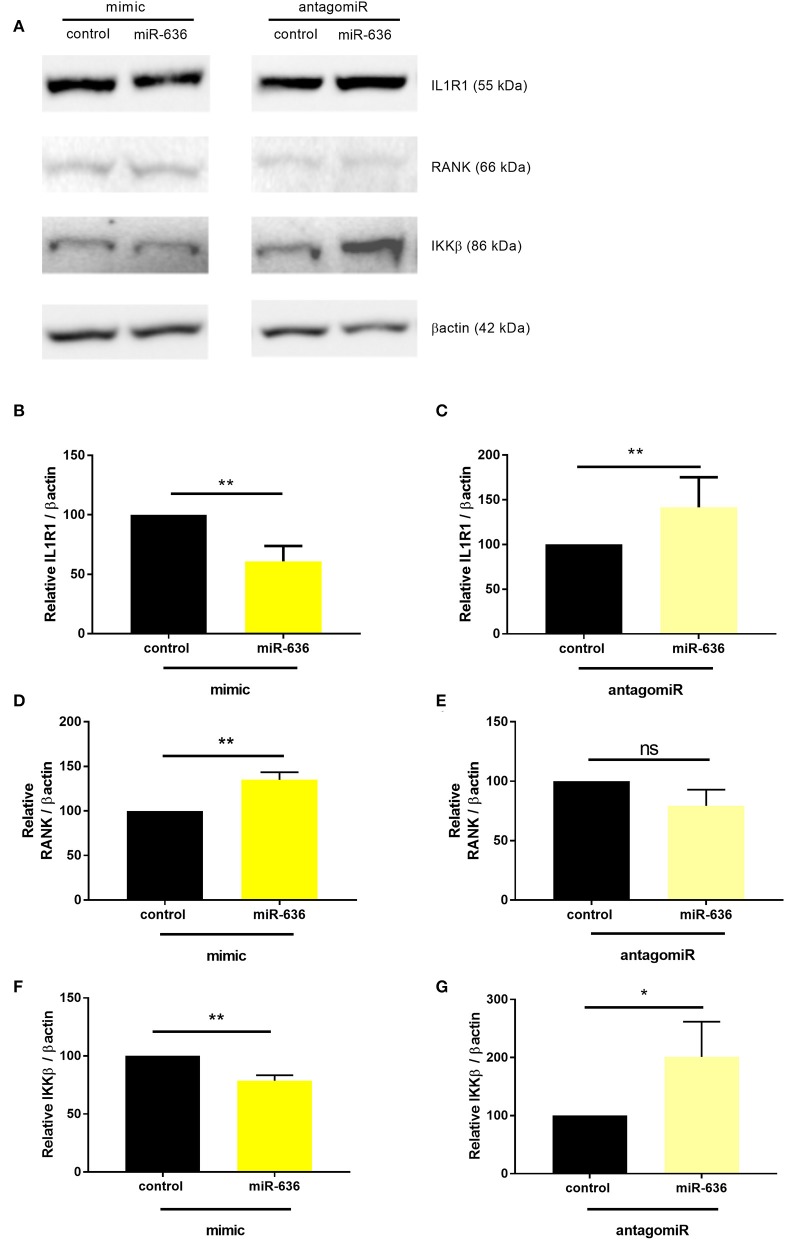
Effect of miR-636 on the protein expression of IL1R1, RANK, and IKKβ. Representative Western blot of protein expression levels **(A)**. Quantification of the protein expression of IL1R1 (**B**, *n* = 5, *p* = 0.0079), RANK (**D**, *n* = 6, *p* = 0.0022), and IKKβ (**F**, *n* = 5, *p* = 0.0079) transfected with a mimic of miR-636 or a mimic control over 48 h. Quantification of IL1R1 protein expression (**C**, *n* = 5, *p* = 0.2063), transfected RANK (**E**, *n* = 6, *p* = 0.3636), and IKKβ (**G**, *n* = 6, *p* = 0.0476) with an antagomiR-636 or antagomiR control over 48 h. A Mann–Whitney test was used to determine significance, ^*^p ≤ 0.05 and ^**^p ≤ 0.01.

Under the same conditions described above, we assessed the protein expression of IL1R1, RANK, and IKKβ (not FAM13A, given that it is not regulated by miR-636) (Figure S2, Figure 4A). In the presence of the miR-636 mimic in CF cells, we observed that IL1R1 protein expression was significantly lower by 33% than that in the presence of the mimic control (Figure 4B). We also observed that the antagomiR-636 significantly downregulate IL1R1 protein expression variation compared to the antagomiR control condition (Figure 4C). In the presence of the miR-636 mimic in CF cells, we observed that RANK protein expression was significantly higher by 35% than that in the mimic control (Figure 4D). Furthermore, antogomiR-636 seemed to decrease RANK protein expression by 20% compared to the antagomiR control condition (Figure 4E). In the presence of the miR-636 mimic in CF cells, we observed that IKKβ protein expression was significantly lower by 22% than that in the mimic control (Figure 4F). Moreover, antagomiR-636 significantly increased IKKβ protein expression by a factor of 2 compared to the antagomiR control condition (Figure 4G). We thereby confirmed that overexpression of miR-636 in CF bronchial epithelial cells leads to reduction in IL1R1 and IKKβ, but increase RANK protein expression.

### miR-636 Regulates NF-κB Pathway Activation and IL-8 and IL-6 Secretions

**Figure 5:**
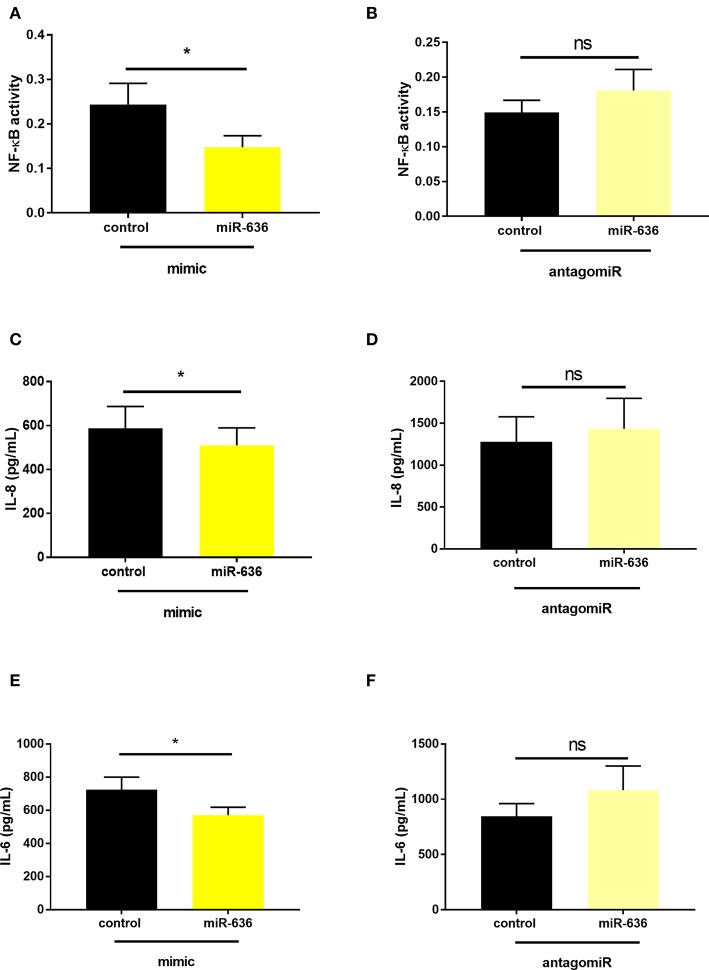
Effect of miR-636 on NF-κB activity and on IL-8 and IL-6 secretion. Quantification of NF-κB activity in CF bronchial epithelial cells (CFBE41o-) transfected with miR-636 mimic or control for 48 h (**A**, *n* = 7, *p* = 0,0156) and antagomiR-636 or antagomiR control for 48 h (**B**, *n* = 3, *p* = 0.4000). Quantification of IL-8 (**C**, *n* = 7, *p* = 0,0246) and IL-6 (**E**, *n* = 5, *p* = 0.0178) secretion in CF bronchial epithelial cells (CFBE41o-) transfected with miR-636 mimic or control mimic for 48 h, and antagomiR-636 or antagomiR control for 48 h (**D**, *n* = 9, *p* = 0.1158; **F**, *n* = 8, *p* = 0.1652) secretion in CF bronchial epithelial cells (CFBE41o-) transfected with antagomiR-636 or antagomiR control for 48 h. S A Mann–Whitney test was used to determine significance, ^*^p ≤ 0.05.

Next, we evaluated the effect of miR-636 on NF-κB activity in the nucleus using the TransAM® method (18). From CF cells transfected with the miR-636 mimic or control and with antagomiR-636 or antagomiR control, we extracted nuclear proteins after 48 h of transfection. NF-κB activity was assessed from the protein extracts through a colorimetric reaction, proportional to NF-κB activity. We observed that the miR-636 mimic significantly decreased NF-κB activity by 38% in CF cells compared to the mimic control (Figure 5A), whereas antagomiR-636 failed to impact NF-κB activity (Figure 5B).

The IL-8 and IL-6 secretions measured by ELISA showed that the miR-636 mimic decreased the secretion of both proteins compared to the mimic control in CF cells (Figures 5C,E). After 48 h, transfection with antagomiR-636 there was no variation in IL-8 and IL-6 secretions compared to the antagomiR control (Figures 5D,F). These experiments demonstrated that NF-κB activity and IL-8 and IL-6 protein expressions are reduced following overexpression of miR-636.

### miR-636 Expression Is Not Modulated by *P. aeruginosa*

**Figure 6:**
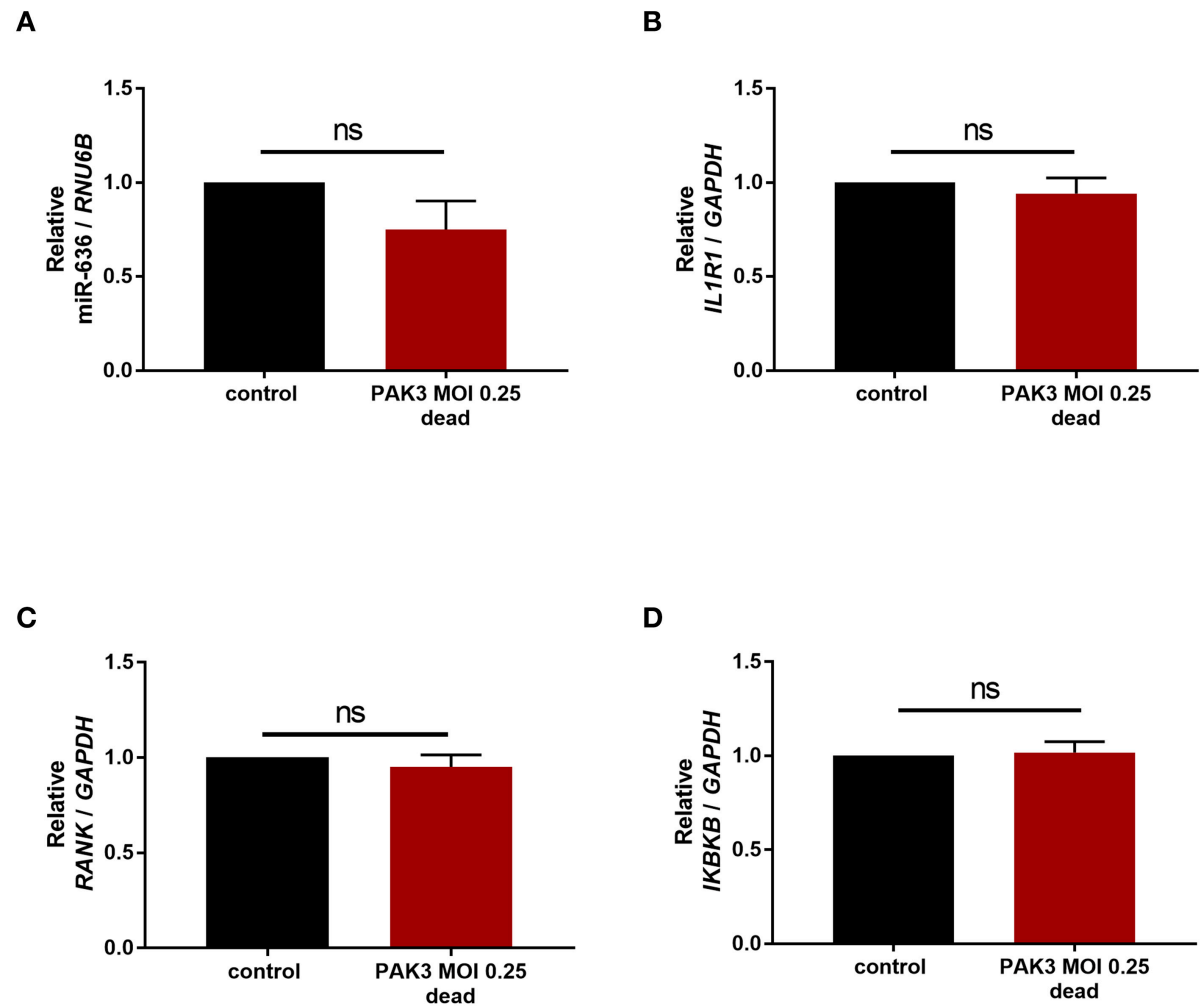
Effect of *Pseudomonas aeruginosa* stimulation on the expression of miR-636, *IL1R1, RANK*, and *IKBKB*. Expression of miR-636 (**A**, *p* = 0.8413), *IL1R1* (**B**, *p* = 0.5476), *RANK* (**C**, *p* = 0.8413), and *IKBKB* (**D**, *p* = 0.8413) was measured in CF cells (CFBE41o-) that were stimulated by *P. aeruginosa* strain PAK heat-killed at an MOI 0.25 or not during 24 h (*n* = 4). A Mann–Whitney test was used to determine significance.

In the airways of CF adult patients, the most common pathogen found is *P. aeruginosa*. We were therefore interested in the possible impact of stimulation by this bacterium on the expression of miR-636. CF cells were stimulated with or without (control) a PAK strain of *P. aeruginosa* at an MOI of 0.25 heat-killed for 24 h. We observed that stimulation by the strain PAK MOI 0.25 heat-killed during 24 h led to no change in *IL1R1, RANK*, or *IKBKB* mRNA or in miR-636 (Figures 6A–D). Surprisingly, we therefore demonstrated that miR-636 expression is not modulated by *P. aeruginosa* in CF bronchial epithelial cells.

### Expression of miR-636 in CF and Non-CF Plasma and Neutrophils

**Figure 7:**
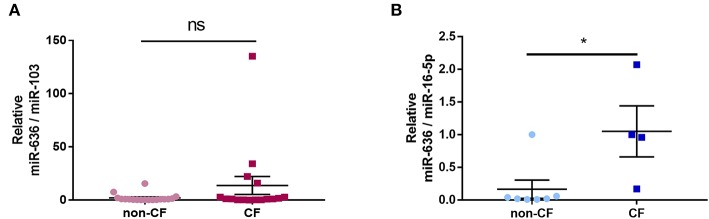
Expression of miR-636 in CF and non-CF plasma and neutrophils. Quantification of miR-636 expression in CF (*n* = 17) and non-CF (*n* = 18) plasma (**A**, *p* = 0.3787) or in CF (*n* = 6) and non-CF (*n* = 7) neutrophils (**B**, *p* = 0.0303). A Mann–Whitney test was used to determine significance, ^*^p ≤ 0.05.

In order to analyse the association between inflammation and miR-636 expression in the plasma of CF patients, we used RT-qPCR to measure the levels of this miRNA in non-CF control and CF patient plasma extracts. We found no significant differential expression of miR-636 in CF patient plasma extracts compared to non-CF plasma control extracts (Figure 7A). However, when we measured the miR-636 levels in non-CF and CF neutrophils, we found a significant increase in miR-636 expression in CF neutrophils compared to non-CF neutrophils (Figure 7B).

## Discussion

In this work, we showed for the first time that miR-636 is able to target key players in the inflammatory cascade and may play a significant role in modulating the inflammatory processes in CF. We demonstrated using functional analysis that this miRNA could modify IL1R1, RANK, and IKKβ expression by a direct interaction on the 3′-UTR mRNA regions. The first articles reporting the dysregulation of miR-636 expression were published in 2011 (21, 22). To our knowledge, in most studies from 2011 to the present day, only miR-636 expression dysregulation has been identified in various pathological contexts, such as myelodysplastic syndrome (21), osteoporosis fractures (23), aortic stenosis (24), dilated cardiomyopathy (25), colorectal tumour (26), colon adenocarcinoma (27), urothelial carcinoma in chronic haemodialysis patients (28), diabetic nephropathy (29), and breast cancer (30), but there have been no such observations regarding CF. Furthermore, only one study has reported a direct link between this miRNA and one of the predicted targets at the 3′-UTR region of GRα (22). Thus, miR-636 expression is associated with various cellular processes, such as the survival, proliferation, inflammation, and repair of DNA.

To identify specific miR-636 targets related to inflammation, we used bioinformatics prediction *in silico* databases that highlighted four actors: *IL1R1, RANK, IKBKB*, and *FAM13A*. We, therefore, focused our study on these four potential targets using functional analysis. In the CF context, we showed that miR-636 specifically binds to the 3′-UTR regions of *IL1R1, RANK*, and *IKBKB*, but not to *FAM13A* mRNA. The specificity of binding is shown using a mutated luciferase plasmid with a 3–5-nucleotide mismatch mutation into the seed region of miR-636. The absence of fixation of miR-636 correlated with sustained luciferase activity. The miRNA interactions led to decrease in the expression of the ILR1 and IKKβ proteins and to increase in the expression of the RANK protein following overexpression of miR-636. Moreover, the overexpression of miR-636 in CF cells causes a significant decrease in the activation of the NF-κB pathway and in the secretion of IL-8 and IL-6—parameters that are well-known to be dysregulated in CF airways (5, 31).

Our study consolidates the results we previously obtained in the small RNAseq experiments of 2018 (18). In fact, we showed overexpression of miR-636 in bronchial epithelial cells from CF patients cultured in ALI compared to those from healthy subjects. We did not observe a significant difference in *IL1R1, RANK*, and *IKBKB* mRNA expression in these same cultures as was the case in the RNAseq results. This is likely due to the fact that it is the protein and not the mRNA expression that is dysregulated by miRNAs in most cases. By measuring the expression levels of different targets in bronchial explants from CF and non-CF patients, we showed no significant increase in miR-636 and *RANK* expression levels, a significant decrease in *IL1R1* expression, significant increases in *IKBKB, FAM13A*, and *IL-8* expression levels, and apparent decrease in *IL-6* mRNA expression in bronchial explants of CF patients compared to those of healthy subjects.

As for many miRNAs, the origin of the dysregulation of miR-636 expression in the CF context is not known and remains to be identified. In another pathological context, a link has been established with adenine nucleotide translocator 2 (ANT2) (32). ANT2—a mitochondrial carrier family 25 member 5—inversely contributes to the translocation of ADP from the cytoplasm to the mitochondrial matrix and to ATP. The suppression of this gene has been shown to induce apoptosis and inhibit tumour growth. Indeed, a decrease in miR-636 expression is associated with cell proliferation via an increase in Ras expression, one of the putative targets of this miRNA. In addition, the suppression of ANT2 restores the expression of miR-636, thus making it possible to reduce the expression of Ras and to inhibit cell proliferation (32) through the PI3K/Akt pathway (33). A decrease in miR-636 is also involved in the recovery from heart failure due to dilated cardiomyopathy (25). Furthermore, in human endothelial cell progenitors, brain-derived neurotrophic factor is able to increase miR-636 expression (34). Interestingly, miR-636 manifests other biological effects; for instance, circulating miR-636 is involved in the elimination of hepatitis C virus (HCV) and also in liver regeneration. miR-636 may constitute a potential therapeutic target for HCV patients with persistent infection despite antiviral treatment (35). Another hypothesis involving miR-636 dysregulation is the modulation of miRNA gene methylation. In fact, a study of miRNA methylation showed that in the context of a weight-loss diet, miR-636 is hypomethylated and overexpressed in the white blood cells of high-responder patients (36). Further investigations are needed to understand the origin of miR-636 dysregulation in the context of CF and the link with CFTR deficiency.

Regarding the implication of miRNAs in the inflammatory process, only two articles have focused on IL-8 secretion regulation by miRNA in CF (37, 38). These articles have demonstrated that miR-93 and miR-17 are implicated in this regulation, but publications are too limited to elucidate the entire system. For example, different proteins involved in the regulation of the IL-8 network could concern the NF-κB pathway, such as the IκBα or the IκB kinases (IKK). In this manner, we have recently shown that miR-199a-3p decreases the expression of IKKβ by direct interaction, hence the activation of the NF-κB pathway and the secretion of IL-8 (18). Recently, others have demonstrated that overexpression of miR-199b decreases the expression of IKKβ-NF-κB, TNF-α, and IL-1β in acute spinal cord injury (39). However, miR-199b is an isoform with a mature sequence identical to miR-199a-3p (40). Here, we showed similar results between the overexpression of miR-636 and the increase of IL-8 secretion levels even when the target was different.

In our study, we demonstrated the direct interaction between miR-636 and *IL1R1, RANK*, and *IKBKB* leading to an indirect effect on IL-8 secretion. Even if the link is evident between IKKβ and IL-8 through the NF-κB pathway, IL1R1 and RANK are proteins that have been shown to be involved in other inflammation-related diseases. For example, IL1R1 shares downstream targets common to toll-like receptor 4 (TLR4) (41). TLR4 plays a pivotal role in innate immunity in its activation of inflammatory signalling pathways (42) in response to various pathogens including viruses, bacteria, and fungi (43). Given that the TLR4 pathway is also dysregulated in the context of CF (6, 44), it would be interesting to determine the impact of miR-636 overexpression. Overexpression of miR-636 inhibiting IL1R1 expression could markedly downregulate the TLR4 signalling pathway not just in CF but also in other diseases, such as epilepsy, in which inactivation of the IL1R1/TLR4 pathway is implicated (45). Activation of TLR4 prevents the translocation of interferon regulatory factor 3 and thus the synthesis of genes, such as *IFN1* required for dendritic cell activation and clearance of CF pathogens (6). The *IL1R1* gene that encodes the receptor whose activation occurs by binding a specific ligand activates the NF-κB pathway, which is widely known as a modulator of the expression of inflammatory and immune genes, such as IL-8 (31, 46–48). It has also been shown that TLR4-activated NF-κB rapidly increases the expression of miR-9 that fine-tunes NF-κB-dependent responses (49). Altogether, these data reveal that the regulation of proteins is highly complex and involves numerous parameters that remain to be elucidated. Such regulation is tightly controlled by transcription factors and also by miRNA.

For the third target, we identified that TNF-α activates the inflammatory pathways by binding to TNF receptors, such as TNFRSF11A, also called RANK, in the context of CF. We showed that miR-636 specifically binds to *RANK* at its 3′-UTR region and thus increases its protein expression. This regulation could be explained by a non-canonical pathway in which microRNAs can up-regulate translation. This mechanism is poorly understood, and in some conditions, miRNAs oscillate between repression and activation (50). RANK acts as an essential regulator in osteoclastogenesis (51, 52) but also controls the organogenesis of lymph nodes (51), intestinal development (53), thymic medullary epithelial cells (54), and the function of the mammary glands during pregnancy (55). Additionally, the RANK system has been demonstrated in the direct regulation of IL-8 in B chronic lymphocytic leukaemia cells (56). Furthermore, one study also reported its involvement in lung cancer cells (11), illustrating its role in the lung. Our study identified the regulation of RANK by miR-636 in CF cells. These results suggest for the first time the direct regulation of RANK at the pulmonary level by a miRNA. As our results demonstrate, overexpression of miR-636 in CF cells leads to a significant decrease in NF-κB pathway activation in CF epithelial cells. We previously identified the overexpression of miR-636 in ALI cells in small RNAseq, but the NF-κB pathway is hyper-activated in the context of CF. We hypothesised that the difference in expression between CF and non-CF could not be sufficient to reverse NF-κB activation and that other miRNAs could be implicated. Additionally, we hypothesise the existence of a long non-coding RNA (lncRNA) in the cell that could block the action of miR-636 on these different targets and could act as a miRNA sponge containing several miRNA binding sequences, as previously described (17). We will use a database to predict the interaction between miRNA and lncRNA (57) to determine, in the context of CF, which lncRNA is overexpressed and be able to block the expression of miR-636. A more thorough understanding of miR-636 will allow us to consider this miRNA as a possible therapeutic target to reduce inflammation in CF patients.

We also showed in our cell model, that stimulation with heat-killed *P. aeruginosa*, the pathogen most commonly found in the airways of CF patients, at an MOI of 0.25 for 24 h, had no effect on the miR-636, *IL1R1, RANK*, and *IKBKB* expression levels. This experiment revealed that the virulence factors are not implicated in this activation. We selected a protracted-time interval to assess the possible impact on the expression of our miRNA, but it is possible that it was too long to observe a significant impact on the expression levels in mRNA. Future studies should consider examining the protein expression levels of IL1R1, RANK, and IKKβ to identify the potential effects of infectious stimulation by living bacteria.

Our results suggest that miR-636 can be considered as a biomarker of inflammation in the blood neutrophils of CF patients but not in the plasma. For example, in combination with other miRNAs (miR-150, miR-145, and miR-223), miR-636 could serve as a biomarker in the blood as shown previously (58) and as a biomarker of renal pathology associated with diabetes, in that miR-636 is expressed in exosomes present in the urine of patients (59). It would be interesting to assess the expression levels of miR-150, miR-145, and miR-223 in the plasma in addition to miR-636 expression. In the prostate context, the decreased expression of miR-636 may be a predictive biomarker (60). We showed that the expression of miR-636 appeared to be higher in the plasma of CF patients and was significantly elevated in the blood neutrophils of CF patients compared to those of healthy subjects. It is likely that in combination with other miRNAs, miR-636 can serve as a biomarker of inflammation in CF patients as previously shown in the plasma (58, 61) and in neutrophils (62).

In addition to basic science implications, our data might be of interest for applied biomedicine because we demonstrated that miR-636 is implicated in the inflammatory process in the CF context to limit IL-8 secretion and chronic neutrophilic lung diseases in CF airways. Moreover, miR-636 present in the neutrophils of CF patients could serve as potential biomarker for detecting early-stage CF airway inflammation.

## Data Availability Statement

All datasets generated for this study are included in the article/Supplementary Material.

## Author Contributions

PB performed and designed the experiments, interpreted results, obtained funding, and wrote the manuscript. TF performed the certain experiments. NR and HC interpreted the certain results. CR and JP provided the neutrophils. OT performed and designed the experiments, interpreted the results, obtained the funding, and wrote the manuscript. All authors reviewed the manuscript.

### Conflict of Interest

The authors declare that the research was conducted in the absence of any commercial or financial relationships that could be construed as a potential conflict of interest.
