# Supplementary material for: miR-636: A Newly-Identified Actor for the Regulation of Pulmonary Inflammation in Cystic Fibrosis
Source: Front Immunol. 2019 Nov 15;10:2643. doi: 10.3389/fimmu.2019.02643 (PMC6874100; doi:10.3389/fimmu.2019.02643)
Supplement: Supplementary file 1 [file Data_Sheet_1.docx]

**SUPPLEMENTARY FIGURES**

**Figure S1: miR‐636 transfection concentration determination. Expression of miR‐636 after transfection of a mimic control or miR‐636 at concentrations of 10, 30, 60, or 100 nM in CF cells (CFBE41o-) (A, n = 1). Expression of miR‑636 after transfection of a mimic control or miR-636 (B, n = 6, p < 0.0001), or antagomiR control or antagomiR-636 (C, n = 6, p=0.0397) at 30 nM in CF cells (CFBE41o-) during 48 h. Student’s t-test was used to determine significance.**

**Figure S2: Expression of *IL1R1* and *RANK* following siRNA transfection. Quantification of *IL1R1* and *RANK* expressions in CF cells (CFBE41o-) after a siRNA transfection at different concentrations (ns: no stimulation, ev: empty vector, 5, 10, 20, 100 nM siRNA) during 48 h (n = 1).**
